# Viral diversity influences T-cell responses to enteric human adenoviruses F40 and F41

**DOI:** 10.1093/ve/veaf098

**Published:** 2025-12-18

**Authors:** Holly M Craven, Jennifer P Hoang, Rookmini Mukhopadhyay, Arnold W Lambisia, Benjamin A C Krishna, Benjamin J Ravenhill, Charles N Agoti, Charlotte J Houldcroft

**Affiliations:** Department of Genetics, Downing Street, University of Cambridge, Cambridge, CB2 3EH, United Kingdom; Roving Researcher Programme, School of Biological Sciences, 17 Mill Lane, University of Cambridge, Cambridge, CB2 1RX, United Kingdom; Department of Genetics, Downing Street, University of Cambridge, Cambridge, CB2 3EH, United Kingdom; Department of Genetics, Downing Street, University of Cambridge, Cambridge, CB2 3EH, United Kingdom; Kenya Medical Research Institute-Wellcome Trust Research Programme, Hospital Road, Kilifi, PO Box 230-80108, Kenya; Department of Medicine, Puddicombe Way, University of Cambridge, Cambridge, CB2 0QQ, United Kingdom; Department of Medicine, Puddicombe Way, University of Cambridge, Cambridge, CB2 0QQ, United Kingdom; Kenya Medical Research Institute-Wellcome Trust Research Programme, Hospital Road, Kilifi, PO Box 230-80108, Kenya; Department of Genetics, Downing Street, University of Cambridge, Cambridge, CB2 3EH, United Kingdom

**Keywords:** epitopes, T cell, adenovirus, evolution

## Abstract

Background: Human enteric species F adenoviruses are a leading cause of diarrhoea-associated paediatric morbidity and mortality worldwide. The cellular immune response (antigen-specific cytotoxic T cells and secreted cytokines) to human adenovirus (HAdV) infection is known to ameliorate symptoms and is critical for viral clearance. We hypothesized that the capsid proteins (hexon and penton) of HAdV-F40 and 41 (F40, F41) are evolving to escape cellular immune responses. Major histocompatibility complex (MHC) binding of viral peptides is a key step in the presentation of peptide–MHC complexes which activate the T-cell receptor and the cytotoxic T-cell response. Methods: Using global HAdV genomic data, we predicted MHC–peptide binding within the hexon and penton proteins of F40 and F41. We focused on MHC Class I alleles common in the UK and Kenya and identified predicted MHC Class I epitopes. Eight predicted epitope pairs from the F41 hexon were synthesized as 15-mer peptides, comparing the wildtype (1970 F41 reference) to the variant (2019–22) sequences. Cellular interferon gamma (IFN-γ) responses to these epitopes were measured in healthy donors using FluoroSpot assays. Results: We identified multiple predicted Class I epitopes shared between HAdV species C and F, but also unique to species F, and epitopes unique to each genotype. We show that IFN-γ and IL-2 (interleukin 2) peripheral blood mononuclear cell (PBMC) responses to HAdV-F are ubiquitous among healthy adult donors from Cambridge, UK. Among predicted Class I epitopes within the F41 hexon, 11/16 peptides elicited donor positive IFN-γ responses from healthy donor PBMC (at least one epitope from seven out of eight peptide pairs). Conclusions: The hexon and penton proteins of HAdV-F-40 and F41 are predicted to contain a number of genotype-specific, but conserved, Class I epitopes which could be used to inform future vaccine design. Using the hexon of F41 as a case study, we show that predicted T-cell epitopes in emergent strains are able to elicit an inflammatory cytokine response from healthy donor PBMC. The role of T-cell recognition in driving enteric adenovirus evolution deserves further consideration.

## Introduction

Adenovirus is a leading cause of illness, hospitalizations, and deaths due to diarrhoea in children under five worldwide (Cohen et al. 2022, Guga et al. 2022). Human mastadenoviruses (HAdVs) are a diverse genus of non-enveloped, double-stranded DNA viruses, of which more than 100 genotypes in seven species (grouped A-G), have been identified. The global burden of acute gastroenteritis (AGE) attributable to HAdVs is significant, with an estimated 75 million cases (Troeger et al. 2018, Lee et al. 2020). Approximately half of the cases of HAdV-associated AGE in children under five are associated with just two genotypes, the only known members of species HAdV-F: 40 and 41 (Liu et al. 2016). Over 35 000 deaths per year are attributable to F40 and F41 in children under five (Cohen et al. 2022). These two genotypes were initially distinguished as a result of their unique serological profiles: neutralizing antibodies to F40 do not neutralize F41, and ‘vice versa’ (De Jong et al. 1983). The two genotypes are 85.5% similar at a genomic level (Götting et al. 2022). In the UK, since at least the 1980s, the majority of HAdV-F AGE is caused by F41 (Cooper et al. 2000), whereas in Kenya, F40 is detected at a ratio of 1:1.2 relative to F41 (Lambisia et al. 2023). No contemporary comparable statistics exist for the UK.

Much of the HAdV genomic diversity is concentrated within the adenovirus capsid genes: hexon, penton, and fibre (Lion and Wold 2021), which are key targets for the adaptive immune response to infection (Sumida et al. 2005, Yu et al. 2013, Hu et al. 2018). It is recognized that escape from neutralizing antibody responses is an important evolutionary driver in genetic diversity of species B (known to cause respiratory and urinary tract infections) and is likely also a driver of high recombination within the capsid genes of HAdV-D species (known to cause ocular, gastrointestinal, and respiratory diseases) (Robinson et al. 2011, 2013, Ismail et al. 2018). However, the impact of evolutionary pressure to escape from T-cell recognition has been less well studied, particularly in enteric adenoviruses. For example, mice with pre-existing T cell and antibody-mediated immunity to adenovirus vectors experienced more pronounced dampening of immune stimulation by HAdV-C5 vectors than mice with passively transferred antibodies alone (Sumida et al. 2004). Additionally, the immune response to HAdV-C5 vectors was decreased by passive transfer of CD8^+^ T cells into naïve mice (Sumida et al. 2004).

Binding of viral peptides and presentation of a peptide-major histocompatibility complex (pMHC) Class I complex is an important step in the activation of the T-cell receptor (TCR) and the CD8^+^ cytotoxic T-cell response (Hennecke and Wiley 2001, Reynisson et al. 2020). Previously identified T-cell epitopes for other adenovirus species have been shown to concentrate in the variable hexon and penton genes (Leen et al. 2004, 2008, Tang et al. 2006, Veltrop-Duits et al. 2006, Hutnick et al. 2010, Mukhopadhyay et al. 2025). We have previously shown that healthy donors make IFN-γ responses (a proxy for CD8^+^ T-cell responses) to both conserved and variable regions of the hexon protein, while IL-2 responses (a proxy for CD4^+^ T-cell responses) are more likely to be towards the conserved domains (Mukhopadhyay et al. 2025), echoing the findings of others (Onion et al. 2007).

We and others have previously described the genetic diversity of F40 and F41 in the UK and Kenya during the periods 2015–22 (UK) and 2013–22 (Kenya) (Lambisia et al. 2023, Maes et al. 2023, Morfopoulou et al. 2023, Reyne et al. 2023). We hypothesized that HAdV-F40 and F41 were under selection pressure to escape from cellular immunity [antigen-specific cytotoxic T cells and secreted cytokines (Lockhart et al. 2022)] and in particular CD8^+^ T-cell recognition, which has been shown to occur for viruses such as Severe acute respiratory syndrome coronavirus 2 (SARS-CoV-2) (Dolton et al. 2022, Stanevich et al. 2023), Epstein-Barr virus (EBV) (Burrows et al. 1996), and influenza A (Machkovech et al. 2015).

In this study, we predicted MHC Class I epitopes within the hexon and penton genes of F40 and 41 sequences from UK and Kenyan samples collected over the course of a decade (~2013–22), relative to the reference sequences for each genotype (isolated in the 1970s). We identified, using FluoroSpot, a group of UK blood donors with robust T-cell responses to species F adenoviruses. We synthesized predicted individual variable epitopes as peptides and used these for T-cell stimulation in FluoroSpot assays. This allowed us to quantify the frequency of T cells producing an interferon gamma (IFN-γ) response to predicted variable epitopes. Finally, we assessed the ability of peripheral blood mononuclear cells (PBMCs) derived from healthy blood donors to make an antiviral response to the diversity of adenovirus F41 hexon variants in current or recent circulation within the UK. We found that healthy blood donors could make an IFN-γ response to at least one epitope from seven out of eight predicted epitope pairs.

## Materials and methods

### Sequence data and alignment

Reference HAdV genome sequences were retrieved from National Center for Biotechnology Information (NCBI) GenBank (https://www.ncbi.nlm.nih.gov/genbank/) on 16 January 2023. DNA coding sequences (CDSs) for the hexon and penton genes were downloaded from GenBank in FASTA format for reference genomes of HAdV-C1 (AC_000017.1), HAdV-C2 (AC_000007.1), HAdV-C5 (AC_000008.1), HAdV-F40 (NC_001454.1), and HAdV-F41 (DQ315364.2). DNA sequences were imported into MEGA11 (Tamura et al. 2021) and translated into protein sequences.

All F40 and F41 complete or partial genome sequences identified from metadata as originating from the UK or Kenya were retrieved from GenBank on 7 February 2023 and downloaded in FASTA format. GenBank was also searched for full-length F40 and F41 hexon and penton sequences without associated whole genome sequences, but none were identified. The DNA CDSs for the hexon and penton were extracted using Multiple alignment program for amino acid or nucleotide sequences (MAFFT) (Katoh et al. 2019) (https://mafft.cbrc.jp/alignment/server/specificregion-last.html), using default settings and ambiguous nucleotides (N) treated as a wildcard. The DNA CDSs from DQ315364.2 (strain Tak) and NC_001454.1 (strain Dugan) were used as references for F41 and F40, respectively. Extracted sequences were downloaded in FASTA format. For a full list of retrieved sequences, see Supplementary Data S1 (GenBank sequences).

All HAdV-F hexon and penton DNA CDSs, including reference sequences, were imported into MEGA11 and translated into protein sequences. Multiple Sequence Comparison by Log-Expectation (MUSCLE) in MEGA11 software with default parameters was used to generate amino acid sequence alignments for the hexon and penton. Alignments were manually corrected where necessary.

### Identification of conserved and variable regions

Conserved and variable regions were identified based on previously published analyses. Hexon: (Crawford-Miksza and Schnurr 1996); penton: (Zubieta et al. 2005).

### Identification of common HLA alleles

Kenyan human leukocyte antigen (HLA) frequencies were retrieved from the Allele Frequency Net Database (Gonzalez-Galarza et al. 2020) on 19 January 2023, using the ‘HLA classical allele frequency search’ tool. Frequencies were derived from a study of Kenyan women of diverse ethnic origin. The locus was restricted to A or B. The population was restricted to ‘Kenya (*n* = 144),’ with the rationale of this population being more representative of nationwide HLA allele frequencies than studies on smaller regions. All other fields were as default. HLA-A and HLA-B alleles were re-classified to two-field resolution and ranked by allele frequency. Since the catalogue of common, intermediate, and well-documented HLA alleles in version 3.0.0 defines a well-documented HLA allele as having ≥5 occurrences in unrelated individuals (Hurley et al. 2020), a cut-off of 3% was chosen, which identified alleles with ≥5 occurrences in a sample size of 144 (Supplementary Data S4 Kenya common HLA; 10 HLA frequencies). Alleles with frequency ≥3% were classed as ‘common’ in Kenya.

UK HLA frequencies were obtained from two studies. One comprised individuals of Caucasian ancestry from the Oxford Biobank (*n* = 5553) (Neville et al. 2017), chosen because of its large sample size. The second comprised individuals from the English blood donor population (*n* = 519) (Davey et al. 2017), including those of non-British descent, chosen to partially recapitulate UK allelic variation owing to ethnic diversity. HLA-A and HLA-B alleles were re-classified to two-field resolution and ranked by allele frequency for each dataset (Supplementary Data S5 UK common HLA; Supplementary Data S10 HLA frequencies). Any allele at frequency ≥3% in either dataset was classed as ‘common’ in the UK to allow for comparison with the Kenyan dataset.

### Prediction of Class I epitopes

Peptide–MHC Class I complexes which may function as epitopes predominantly for CD8^+^ T cells (Hennecke and Wiley 2001) were predicted using NetMHCpan-4.1 (Reynisson et al. 2020). Predictions were restricted to 9-mers. Strong binders (SBs) were defined by %Rank<0.5; weak binders (WBs) were defined by %Rank<2.

HAdV-C1 (AC_000017.1), HAdV-C2 (AC_000007.1), and HAdV-C5 (AC_000008.1) hexon- and penton-derived epitopes were predicted, with HLA-A and HLA-B alleles selected according to HLA restrictions of experimentally validated epitopes identified by literature review (Supplementary Data S2 Published epitopes and Supplementary Data S3). Predictions were cross-checked with experimentally validated epitopes (except for an HLA-B^*^63-restricted hexon-derived epitope, due to HLA-B^*^63 being unavailable in the NetMHCpan-4.1 database). For experimentally validated epitopes longer than nine residues, successful prediction was defined as prediction of any nine contiguous residues within that epitope.

C5 (AC_000008.1), F40 (NC_001454.1), and F41 (DQ315364.2) hexon- and penton-derived epitopes were predicted for HLA-A and HLA-B alleles identified as common in Kenya and/or the UK.

### Characterization of epitope variation

For predicted F40 (NC_001454.1) and F41 (DQ315364.2) SB epitopes that were not shared by C5, intratypic variation was identified by manual inspection of the hexon/penton sequence alignments. Where variation was found, binding of the variant sequence(s) to HLA-A and HLA-B alleles identified as common in Kenya and/or the UK was predicted using NetMHCpan-4.1 (settings as above). The change in total allele frequency of tested HLA alleles predicted to be SBs, WBs, or NBs to the variant sequence compared to the corresponding reference sequence (NC_001454.1/DQ315364.2) was calculated in each case, using HLA frequencies from the geographic region(s) where the predicted variant epitope was identified. Both common Kenyan and UK HLA allele frequencies were considered, independent of the country-of-origin of the variant strain.

### Peptide synthesis and preparation of peptides

A custom library spanning the F41 hexon protein was synthesized by GenScript (Oxford, UK) and reconstituted into a hexon peptide pool as previously described (Mukhopadhyay et al. 2025). The F41 amino acid sequence of strain Tak (DQ315364.2) was used as the ‘wildtype’ sequence. Variable amino acid sequences predicted to contain potential T-cell epitopes (see previous section) were also synthesized. These ‘variant’ sequences, Supplementary Data S11 (Variant epitopes), were derived from Supplementary Data S1 (GenBank sequences) from the UK dating between 2019 and 2023 for eight peptides. Individual peptides were used to stimulate PBMC at a concentration of 40 μg/ml/peptide.

### Ethics and donor cohort information

Ethical approval for work on samples from healthy human donors was granted for project 315068 ‘Understanding humoral and cellular immune responses to DNA viruses in healthy blood donors’ by the HRA and Health and Care Research Wales (REC reference: 22/WA/0162). Cells were collected by the National Health Service Blood and Transplant Service in Cambridge, UK and supplied for non-clinical use.

### PBMC isolation

PBMC isolation from leukocyte reduction cones was performed as previously described (Mukhopadhyay et al. 2025). Samples were stored frozen in liquid nitrogen for batch analysis.

### Detection of cytokine production by FluoroSpot

Cytokine production by PBMC was quantified using a dual FluoroSpot Flex kit which was specific for human IFN-γ [capture mAb (1-D1K); BAM ((benzo[a]acridin-12-yl)methyl)-conjugated detection mAb (7-B6–1); anti-BAM-490] and human IL-2 [capture mAbs (MT2A91/2C95); biotinylated detection mAb (MT8G10); SA-550] (X-01A02B-10, Mabtech, Sweden).

Plates were prepared and PBMC samples thawed, treated with Benzonase Nuclease (Merck), and counted as previously described (Mukhopadhyay et al. 2025). Briefly, 1.5–2.5 × 10e^5^ cells/well of donor PBMCs in TexMACS (Miltenyi Biotech, UK) were pipetted into 96 well FluoroSpot plates, coated with either anti-human IFN-γ or anti-human IL-2. Peptide and protein were added in concentrations defined in Table 1, and the final volume adjusted to 150 μl/well. ImmunoCult™ Human CD3/CD28 T-Cell Activator (STEMCELL Technologies, UK) was used as a positive control. TexMACS media (Miltenyi Biotec, UK) with 0.01% dimethyl sulfoxide (DMSO) (Sigma-Aldrich, UK) was used as the negative control. All stimulations were carried out in triplicate. Samples were left to incubate for 40 h before antibody detection was performed according to manufacturer’s instructions. Plates were dried in a 37°C incubator before acquisition using an AID iSpot reader (Advanced Imaging Devices, Germany). Plates were manually quality controlled, IFN-γ data were normalized and analysed as in Houldcroft et al. (2020), and IL-2 data were normalized and analysed as in Krishna et al. (2024).

**Table 1:** Identity, supplier, genotype, and concentration of peptide and viral proteins

Name	Supplier	Adenovirus species and type	Final concentration
Custom HAdV F41 hexon pool	GenScript	F: 41	2 μg/ml/peptide
Individual F41 peptides	GenScript	F: 41	40 μg/ml/peptide
Native adenovirus type 40 protein (ab123997)	Abcam	F: 40	3.3 μg/ml^a^
Adenovirus type 40 hexon protein (NAT41552)	The Native Antigen Company	F: 40	3.3 μg/ml

a Native adenovirus F40 protein was used as a final concentration of 3.3 μg/ml (Sinclair et al. 2004).

### Statistical analysis

Statistical analyses were performed and data visualized in GraphPad Prism v10.4.1.

## Results

### Identification of common Kenyan and UK HLA alleles

**Figure 1:**
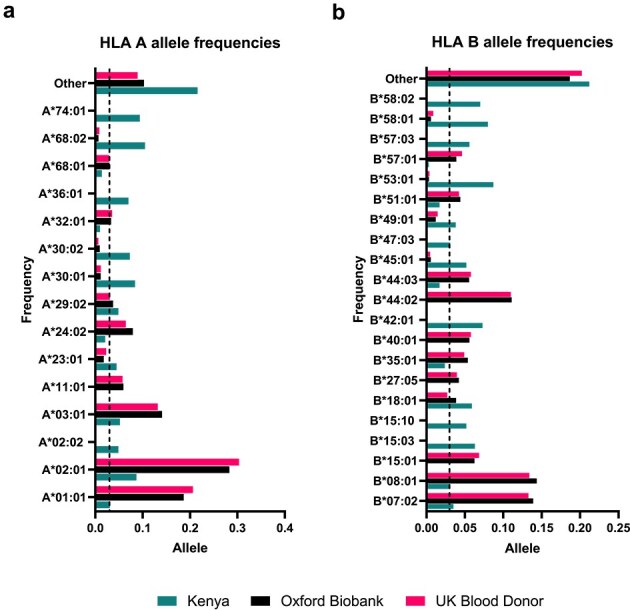
HLA allele frequency distributions for common HLA-A (a) and HLA-B (b) alleles in Kenyan and UK populations; HLA allele frequencies were obtained from UK-based studies using data from the Oxford Biobank (*n* = 5553, shown in black) (Neville et al. 2017), a study of the UK blood donor population (*n* = 519, shown in pink) (Davey et al. 2017); Kenyan frequencies were derived from a study of donors of diverse ethnic origin from the Allele Frequency Net Database (*n* = 144, shown in teal) (Gonzalez-Galarza et al. 2020); HLA alleles with a frequency of ≥0.03 in one or more of these three studies are shown; black dashed lines mark the 0.03 threshold used to define common HLA alleles (Hurley et al. 2020).

A single Kenyan dataset was used to identify common (defined as alleles with frequency ≥3%) Kenyan alleles, while two datasets were used for UK alleles (see methods). HLA-A and HLA-B allele frequencies were largely conserved albeit differing in frequency, and the two UK datasets were largely in accordance (Fig. 1). There were 8 (UK) and 11 (Kenyan) HLA-A alleles identified as common (Fig. 1a), of which 4 alleles (A^*^01:01, A^*^02:01, A^*^03:01, A^*^29:02) were conserved between groups. Frequency largely varied between groups within conserved alleles; however, a greater frequency of common alleles was generally observed in UK datasets. The largest variation in allele frequency proportions between the UK group was observed in alleles A^*^01:01 (0.02 difference in frequency between the two UK datasets) and A^*^02:01 (0.02 difference in frequency between the two UK datasets). However, these alleles were also the two most frequent alleles in the UK datasets, whereas in the Kenyan samples, allele A^*^68:02 had the greatest frequency. The Kenyan group had a high frequency of ‘other’ alleles that was greater than individually observed common alleles.

Similar patterns were observed for HLA-B alleles in both Kenyan and UK samples (Fig. 1b). A total of 21 alleles were recorded as ‘common’: 13 in Kenyan data and 11 in UK. Interestingly, there was a single allele that was not concordant between the two UK datasets: B^*^18:01. On average, there was a lower frequency observed across a wider spread of alleles with the greatest frequency <0.15 for both UK and Kenyan data. In all datasets, there is a large frequency (0.2) of alleles in the ‘other’ category, indicating greater spread compared to HLA-A.

### Prediction of experimentally validated HAdV Class I epitopes by NetMHCpan-4.1

To evaluate the ability of NetMHCpan-4.1 to predict HAdV pMHC complexes which are Class I epitopes, it was determined whether NetMHCpan-4.1 could successfully identify experimentally validated Class I epitopes of HAdV-C. From a literature review, 29 experimentally validated Class I epitopes and their respective HLA restrictions were identified for C5, 21 of which were hexon- or penton-derived (Supplementary Data S2. Published epitopes). Over 70% (12/17) hexon-derived and 100% (3/3) penton-derived epitopes were identified as SBs or WBs by NetMHCpan-4.1 for C5 (Table 2). Results were consistent for genotypes C1 and C2 where epitopes were shared. The epitopes which NetMHCpan-4.1 failed to predict were not concentrated in any particular region of the hexon protein. It was concluded that, while not all epitopes could be identified, NetMHCpan-4.1 was a suitable tool for HAdV hexon- and penton-derived Class I epitope prediction.

**Table 2:** Validation of prediction of experimentally identified T-cell epitopes

HLA restriction	HAdV species tested	HAdV protein	T-cell epitope sequence	Amino acid positions^a^	Predicted binding strength	Successfully predicted by NetMHCpan-4.1?
HLA-A^*^01	C2, C5	Hexon	TDLGQNLLY^b^	886–894	SB	Yes
HLA‐A^*^01	C2, C5	Hexon	LTDLGQNLLY^b^	885–894	SB	Yes
HLA-A^*^02	C5	Hexon	TFYLNHTFKKV	711–721	SB	Yes
HLA-A^*^02	C5	Hexon	YVLFEVFDVV	917–926	SB	Yes
HLA-A^*^02	C5	Hexon	LLYANSAHAL	892–901	SB	Yes
HLA-A^*^02	C5	Hexon	GLRYRSMLL	542–550	WB^c^	No
HLA-A^*^02:01	C1, C2, C5	Hexon	TLLYVLFEV	914–922	SB	Yes
HLA-A^*^24	C2, C5	Hexon	TYFSLNNKF	37–45	SB	Yes
HLA-B^*^07	C1, C2, C5	Hexon	KPYSGTAYNSL	114–124	N/A	No
HLA-B^*^07	C5	Hexon	FRKDVNMVL	585–593	SB^c^	No
HLA-B^*^07, HLA-B^*^35	C1, C2, C5	Hexon	MPNRPNYIAF	320–329	WB	Yes
HLA-B^*^08:01	C1, C2, C5	Hexon	DLQDRNTEL	360–368	SB	Yes
HLA-B^*^13, HLA-B^*^49	C5	Hexon	LFEVFDVVRV	919–928	WB	Yes
HLA-B^*^35, HLA-B^*^53	C5	Hexon	IPYLDGTFY	705–713	SB	Yes
HLA-B^*^35:01	C5	Hexon	IPFSSNFMSM	873–882	WB	No
HLA-B^*^52	C5	Hexon	ETYFSLNNKF	36–45	SB	No
HLA-B^*^53	C5	Hexon	LPGSYTYEW	575–583	WB	Yes
HLA-A^*^01:01	C1, C2, C5	Penton	STDVASLNY	76–84	SB	Yes
HLA-A^*^02:01	C1, C2, C5	Penton	ILHTNMPNV	126–134	SB	Yes
HLA-B^*^08:01	C1, C2, C5	Penton	DSKKRSYNL	385–393	SB	Yes

a Amino acid positions are relative to C5 sequence.

b The same 9-mer was predicted for these epitope C Predicted strong or weak binder of different HLA type to experimentally validated HLA types

### Prediction of cross-adenovirus-species T-cell epitopes

NetMHCpan4.1 was used to predict hexon- and penton-derived T-cell epitopes for reference strains of HAdVs C5, F40 (Dugan), and F41 (Tak) for all common Kenyan and UK HLA alleles (see Supplementary Data S4 and S5). Consistent with experimental studies, more epitopes were predicted for the hexon (Supplementary Data S6) than the penton (Supplementary Data S7). Predicted SBs were categorized as being unique to F40, unique to F41, shared by F40 and F41 only (shared HAdV-F epitopes), or shared by all three HAdVs (Supplementary Data S6 and S7). No predicted SBs were unique to C5. Only shared F epitopes and unique F40/F41 epitopes were taken forward for intratypic variation analysis (Supplementary Data S8), since epitopes shared across HAdV species are unlikely to show intratypic variation.

### Characterization of intratypic variation within predicted HAdV-F epitopes

The reference sequences of F40 and F41 have 85.5% genomic similarity (Götting et al. 2022). The average nucleotide identity values for the hexon (78.2%) and penton (79.5%) genes, compared to the short (95%) and long (91.4%) fibre genes (Supplementary Methods) show that much of the difference between genotypes is driven by the variable regions of the hexon and penton (in line with other, more comprehensive studies of human adenoviruses) (Robinson et al. 2013, Ismail et al. 2018).

To assess the intratypic variation in predicted epitopes of F40 and F41, amino acid alignments were generated using all identified hexon and penton sequences from F40 and F41 strains originating from Kenya or the UK, and those of the first sequenced F types (F41 Tak-DQ315264.2 and F40 Dugan-NC001454.1) (Supplementary Data S1). Mutation positions were defined relative to these alignments. UK strains spanned 2015–22; Kenyan strains spanned 2013–22. For F41, 38 UK hexon, 8 Kenyan hexon, 38 UK penton, and 33 Kenyan penton sequences were included. The lower number of Kenyan hexon sequences reflected missing sequence data in hexon-coding regions precluding analysis. For F40, 29 Kenyan hexon sequences and 28 Kenyan penton sequences were identified. No UK F40 sequences were available.

**Figure 2:**
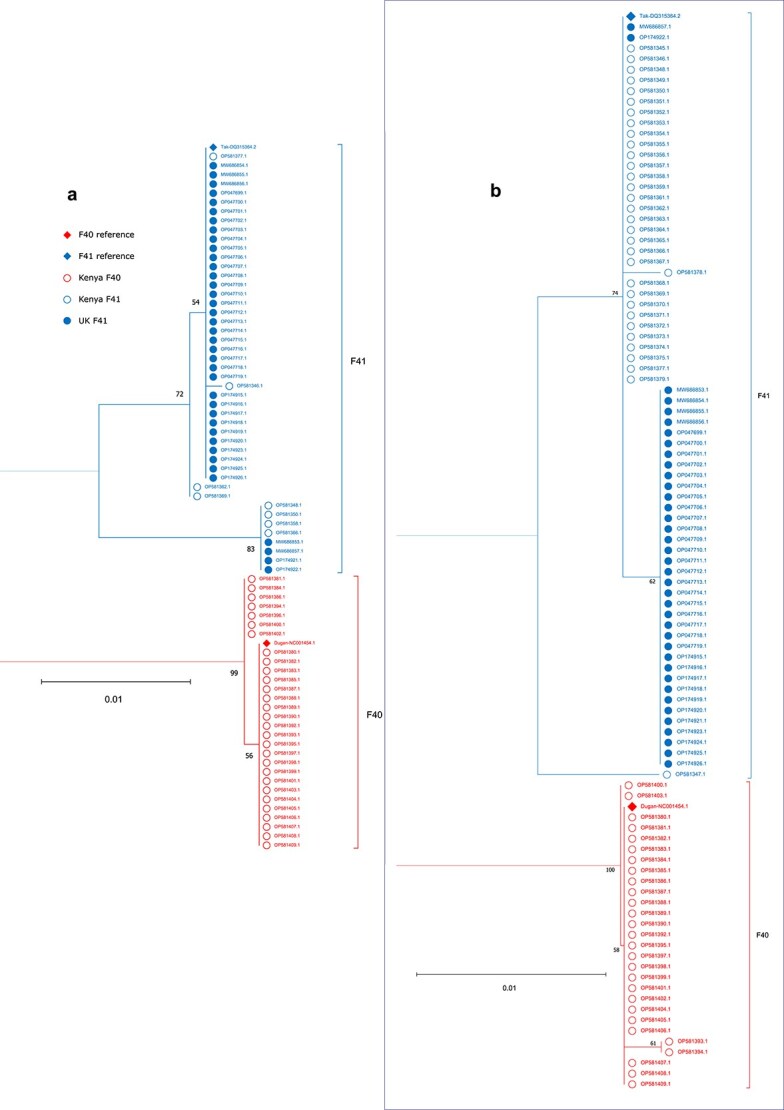
Phylogenetic trees for HAdV-F hexon (a) and penton (b) amino acid sequences; the root and total distance separating the F40 and F41 types are not shown due to the low intertypic sequence identity relative to intratypic sequence identity; F41 sequences are shown in blue; F40 sequences are shown in red; the two reference sequences (F41 Tak-DQ315264.2 and F40 Dugan-NC001454.1) are marked with a filled rhombus at the tip point followed by accession number; sequences derived from UK samples are marked with a filled circle at the tip point followed by accession number; sequences derived from Kenyan samples are marked with an unfilled circle at the tip point followed by accession number; bootstrap support values are shown in black; amino acid sequences were aligned using MUSCLE in MEGA11; maximum likelihood trees were constructed using the amino acid substitution model WAG in MEGA11 (partial deletion, 80% site coverage cut-off).

Phylogenetic analyses at the amino acid level (Fig. 2) revealed that for the hexon (A) and penton (B), F40 and F41 formed two distinct clusters, reflecting deep intertypic divergence relative to intratypic variation [consistent with the lack of intertypic HAdV-F recombination identified by DNA-level phylogenetic analyses (Götting et al. 2022)]. Within F41, hexon and penton sequences tended to cluster by country, suggestive of protein-level geographic variation. Within each type, short branch lengths indicated high conservation of the hexon and penton amino acid sequences. This included Dugan and Tak, despite their isolation in 1979 and 1973, respectively (De Jong et al. 1983).

Of the F41 unique SBs, 12 predicted hexon epitopes and 7 predicted penton epitopes showed intratypic variation (Supplementary Data S9). These contained only amino acid substitutions, except the hexon-derived epitope QTWTADDNY, where variant sequences displayed three amino acid substitutions (Q413G, T414N, and N421T), an amino acid insertion (insN419), or all four changes in combination. Seventeen F41 epitopes across the hexon and penton showed variation in UK sequences, while nine showed variation in Kenyan sequences. However, a number of epitopes were not well covered by the Kenyan sequencing data, so there may be unsampled variation within predicted epitopes among Kenyan F41 strains. F40 appeared to show greater amino acid conservation than F41, with four predicted F40 hexon epitopes and one predicted F40 penton epitope showing intratypic variation. The four variable hexon epitopes overlapped the same single-residue substitution (A254T), while the variable penton epitope harboured a single-residue deletion in a different strain (A305del). However, there is likely unascertained F40 diversity, owing to the lack of available UK sequences and incomplete Kenyan sequencing data. No shared HAdV-F epitopes showed intratypic variation.

**Figure 3:**
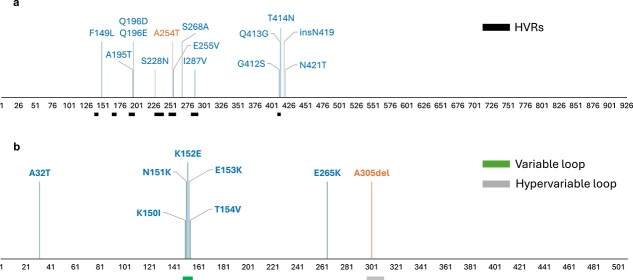
HAdV-F40 and HAdV-F41 variable epitope positions in the hexon (a) and penton (b) proteins; the horizontal axes indicate amino acid positions according to the multiple sequence alignments; vertical lines indicate positions at which intratypic variation was identified in one or multiple predicted epitopes and are labelled with the corresponding mutation(s); F41 mutations are shown in blue; F40 mutations are shown in orange; the hexon hypervariable regions (HVRs) are indicated by black bars (left to right: HVR1 to HVR7) (Crawford-Miksza and Schnurr 1996); the penton variable loop region is indicated by a green bar and the penton hypervariable loop region is indicated by a grey bar (Zubieta et al. 2005); vertical line heights have been varied for clarity and do not indicate the number or type of substitution.

Mutations within predicted HAdV-F epitopes typically resided in or around previously characterized variable regions of the hexon and penton (Fig. 3). All mutations identified within predicted HAdV-F hexon epitopes resided within or near the hexon HVRs, and most predicted HAdV-F penton epitopes resided in the variable or hypervariable loops. Epitopes showing no intratypic variation (where sequence data was available for all strains) were generally located outside variable regions, though there were some exceptions (Supplementary Data S8).

### Prediction of variant epitope binding mediated by common Kenyan and UK HLA alleles

To assess how intratypic variation may affect immune recognition, the total allele frequencies of tested HLA alleles predicted by NetMHCpan-4.1 to be SBs, WBs, or non-binders (NBs) for each variant epitope were calculated for the geographic region(s) where the variant epitope was identified. Frequencies were compared to those for corresponding reference epitopes (Supplementary Data S9). All identified mutations except I287V were associated with predicted binding changes for at least one epitope. In general, the total frequency of common HLA alleles predicted to be SBs decreased or remained unchanged upon mutation, though there were exceptions. The change in WB allele frequency was more variable, partly due to the counteracting contributions of SBs becoming WBs and WBs becoming NBs.

### T-cell responses to HAdV-F40 lysate and F41 hexon

We have previously demonstrated that cellular immune responses to the F41 hexon are widespread and frequent in healthy blood donors (Mukhopadhyay et al. 2025). F41 is more frequently detected in clinical and wastewater samples in the UK and Ireland than in Kenya (Lambisia et al. 2023, Maes et al. 2023, Martin et al. 2023). We therefore wished to compare the cellular immune responses of the same cohort of blood donors from England to see if IFN-γ and/or IL-2 responses to F40 were less frequent than responses to F41.

We compared responses to the hexon protein of F41 and total F40 viral lysate, using dual-coloured FluoroSpot (Supplementary Fig. S1). There was no statistically significant difference (two-way Analysis of Variance (ANOVA); Tukey’s multiple comparisons test) in the frequency of PBMC IFN-γ (*P* = .49) or IL2 (*P* = .97) responses to F40 viral lysate and F41 hexon in a cohort of 13 healthy adult blood donors from Cambridge, UK. Based on positivity thresholds from adenovirus-vectored vaccine recipients (*n* = 13) (Mukhopadhyay et al. 2025), 92% of donors made a positive IFN-γ and 100% of donors made a positive IL2 response to F41; applying the same cut-offs to F40 viral lysate, 83% (*n* = 12) of donors made positive IFN-γ and IL2 responses.

### T-cell responses to F41 hexon variable epitopes

**Figure 4:**
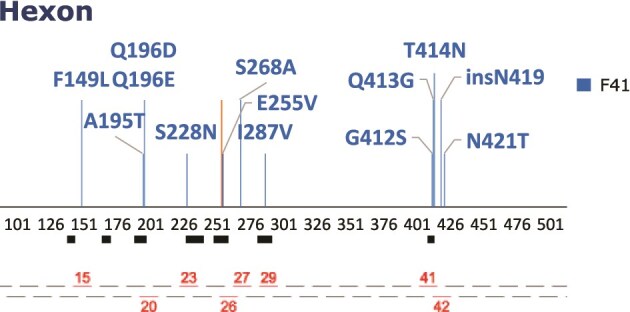
Diagram showing the location of predicted variable epitopes within the F41 hexon (truncated to amino acid positions 110–490) and the location of synthetic 15-mer peptide pairs spanning these amino acid substitutions; peptide pairs are numbered sequentially along the hexon amino acid sequence; filled boxes represent the seven hyper variable domains within the hexon protein sequence (Crawford-Miksza and Schnurr 1996); peptide sequences spanning the highlighted positions are shown in Table 3.

All donors made an IFN-γ and/or IL-2 PBMC response to either F40 or F41 (Supplementary Fig. S1). However, the F40 protein and F41 hexon peptide pool are derived from HAdV-F strains Dugan and Tak, originally isolated in the 1970s (De Jong et al. 1983). Phylogenetic analysis places the F41 reference Tak in F41 lineage 1 (Götting et al. 2022), a lineage which has not been detected in the UK (Maes et al. 2023). We therefore investigated whether changes in predicted T-cell epitope sites within the F41 hexon, which have accumulated between the 1970s and 2019 onwards, have led to changes in immune responses to these regions of the hexon protein. The pMHC binding predictions are based on Class I HLA types; therefore we used IFN-γ as a proxy for successful presentation to, and antiviral response from, CD8+ T cells (Slifka and Whitton 2000). IFN-γ responses were measured by FluoroSpot. Total PBMCs were stimulated with eight pairs of 15-mer peptides, each pair containing a ‘wild type (WT)’ sequence synthesized to match the reference sequence (1970s) and the ‘variant’ sequence synthesized based on sequences from F41 genomes derived from patients from 2019 onwards. Peptide pair locations are shown in Fig. 4 and sequences in Table 3.

**Table 3:** Location and sequences of wild type and variant peptides

Peptide number	Wild type sequence	Variant sequence
15	IKVRGQAPFIGTNIN	IKVRGQAPLIGTNIN
20	EVGAAQKVAGRVLKD	EVGATDKVAGRVLKD
23	NEKGGQASLITNGTD	NEKGGQANLITNGTD
26	STPNEPKAVLYAENV	STPNVPKAVLYAENV
27	YAENVSIEAPDTHLV	YAENVAIEAPDTHLV
29	AQGTISSADLLTQQA	AQGTVSSADLLTQQA
41	TYSGIKANGQTWTAD	TYSGIKANSGNWTAD
42	TWTADDNYADRGAEI	NWTADNDTYADRGAE

**Figure 5:**
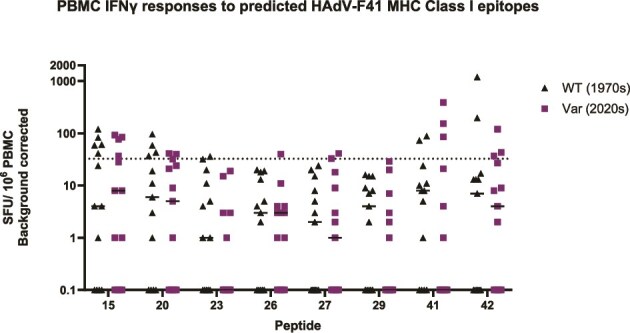
Analysis of AdV-specific IFN-γ FluoroSpot responses to individual peptide stimulation; responses are calculated as spot-forming units (SFUs) per 10e6 PBMC (background corrected); the dotted line indicates the boundary between positive and negative responses (Mukhopadhyay et al. 2025); WT: wild type 1970s reference sequence (black triangles); Var: 2019–22 variant sequence (purple squares).

The FluoroSpot results show that in seven out of eight pairs, at least one predicted Class I epitope elicited an IFN-γ response greater than 32.5 spot-forming unit (SFU) in at least one donor (*n* = 13) (Fig. 5). The peptide to which the highest proportion of donors made a positive IFN-γ response (≥32.5 SFU) was the WT/reference for peptide 15 (38%), located between HVRs 1 and 2. Some donors did not make an above background response to the Class I epitopes in Table 3. As donors were randomly selected rather than selected by HLA type, they may not carry the HLA types predicted to successfully present these Class I epitopes.

**Figure 6:**
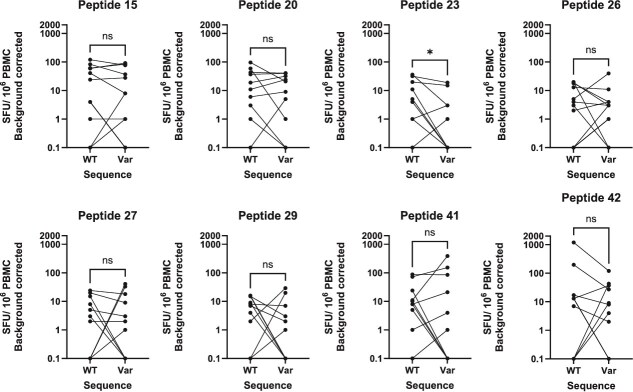
Analysis of paired within-donor PBMC IFN-γ responses to F41 hexon individual peptide stimulation; PBMCs from healthy blood donors were stimulated in triplicate with individual synthetic peptides representing predicted epitopes within the F40 hexon protein which have mutated between the 1970s and 2019–22; mean IFN-γ responses were compared for each donor between pairs of peptides; WT: wild type 1970s reference sequence; Var: 2019–22 variant sequence; responses are calculated as spot-forming units (SFUs) per 10e6 PBMC (background corrected); significance determined by Wilcoxon matched-pairs signed rank test. Key: ^*^P<.05.

SARS-CoV-2, among other viruses, has been shown to be under evolutionary pressure to escape from both individual and population-level CD8^+^ T-cell-driven immunity (Motozono et al. 2021, Stanevich et al. 2023). We hypothesized that HAdV-F is under similar pressure, but acting on longer timescales than SARS-CoV-2. By comparing changes to each peptide within individuals, we can see that no peptide exhibits universal escape from immune recognition, nor universal gain of recognition, although mean IFN-γ responses to peptide 23 were lower (*P* = .03, Wilcoxon matched-pairs signed rank test) (Fig. 6; Supplementary Fig. S2).

## Discussion

HAdV hexon HVRs have previously been identified as important in neutralizing antibody escape (Roberts et al. 2006, Robinson et al. 2013). The results of this study suggest that they are also of interest in escape from CD8^+^ T-cell recognition, ‘via’ MHC Class I binding, since mutations within predicted CD8^+^ T-cell epitopes cluster around the hexon HVRs. The penton variable and hypervariable loops were similarly identified as potentially important in CD8^+^ T-cell escape. For example, we predict A305del to confer partial immune escape at that epitope in a Kenyan F40 strain. Penton alignments by Zubieta et al. (2005) indicate that this position coincides with the highly conserved arginylglycylaspartic acid (RGD) motif in the penton hypervariable loop of other HAdVs. HAdV-F does not possess an RGD motif, reflecting its lack of reliance on cellular integrins for gastrointestinal cell infection (Albinsson and Kidd 1999). Instead, F40 has an RGAD motif and F41 has an IGDD motif (Albinsson and Kidd 1999) so A305del may have fewer fitness effects in HAdV-F than mutations at this position in other HAdVs. This highlights how the unusual properties of HAdV-F (i.e. its enteric tropism) may permit unique mechanisms for immune escape.

Phylogenetic analyses of the hexon and penton reveal high intratypic amino acid conservation between the HAdV-F40 and F41 strains circulating over the last 10 years, and the Dugan and Tak reference strains despite Dugan and Tak being isolated in the 1970s (De Jong et al. 1983). This suggests either a relatively slow mutation rate (as expected in dsDNA viruses) or strong functional constraints on the conserved regions of these proteins. Similar patterns of long-term conservation have been shown in species C HAdV (Dhingra et al. 2019). This study has identified at least one predicted hexon- or penton-derived SB epitope conserved between C5, F40, and F41 for all common Kenyan and UK HLA alleles (Supplementary Data S6), suggesting Class I epitopes can be shared across multiple HAdV species (Gardner et al. 2024, Mukhopadhyay et al. 2025). The use of conserved sequences may facilitate the development of a pan-HAdV vaccine which induces cellular immune responses (Wang et al. 2023, Gardner et al. 2024). Intratypic variation was identified in predicted unique F40 SBs and unique F41 SBs, but not shared HAdV-F SBs, suggesting shared epitopes may reside in regions under evolutionary constraint, precluding mutations from facilitating escape from cellular immunity.

Experimental evolution and deep sequencing of HAdV-C5 found that, *in vitro*, different regions of the adenovirus genome mutate at approximately the same rate, suggesting that selection pressure is the key factor differentiating the conserved and hypervariable regions of the hexon and penton proteins (Risso-Ballester et al. 2016) with homologous recombination within these regions providing a further source of diversity (Robinson et al. 2013). The N and C terminal domains of the HAdV-F41 hexon are highly similar to the hexons of species C, a functional constraint to allow the bases of individual hexon proteins to remain in contact with one another. Changes are concentrated in the HVRs on the hexon surface, and seem penalized elsewhere in the protein because they disrupt the structure and function of the protein (Toogood and Hay 1988, Zubieta et al. 2005).

Escape from adaptive immunity is known to be an important factor in the evolution of HAdV species B and D (Robinson et al. 2011, 2013, Ismail et al. 2018), but much less is known about the evolutionary consequences of cellular immunity in driving adenovirus mutation and recombination through diversifying selection. This is true particularly within species F, where there is no evidence for intertypic recombination between the two known types (Götting et al. 2022). The only representative of species E, HAdV-4, has two phylogroups and there is speculation that phylogroup II may be spreading more successfully than phylogroup I (Kajon et al. 2018) in part because a phylogroup I E4 strain is the basis of the US military adenovirus vaccine, and thus non-vaccine-like E4 strains have a selective advantage, even though all E4 strains remain a single serotype regardless of phylogroup (Gonzalez et al. 2019). This suggests that neutralizing antibody escape alone cannot be the only source of selection acting on human adenoviruses, particularly those with no other genotype members available for recombination.

In the context of resurgent post Coronavirus disease 2019 (COVID-19) pandemic F41 circulation (Maes et al. 2023, Reyne et al. 2023), it is important to know whether extant lineages 2A and 2B have increased immune evasiveness. This study demonstrates that computationally predicted Class I epitopes can elicit antiviral IFN-γ responses from healthy adult blood donors; and that while no predicted epitope was universally immune evasive in our cohort, individual regions of the F41 hexon have mutated over the last ~50 years and these mutations have functional consequences for the magnitude of the IFN-γ response within individual donors. Future work will explore the impact of antigenic variation on MHC Class II presentation and CD4^+^ T-cell responses (Bowyer et al. 2018), and explore a broader range of antiviral cytokine responses to enteric adenoviruses (Altosole et al. 2023, Gardner et al. 2024, Murray et al. 2025). Further analyses will be required to confirm the antiviral effector functions of T cells targeting the epitopes we have identified.

### Limitations to this study

MHC binding and presentation of peptides to CD8^+^ T cells are not the only factor influencing T-cell response. In addition to the anchor motifs at positions 2 and 9, Class I epitopes comprise TCR contact residues, which vary in position between 9-mers (Rudolph et al. 2006). TCR contact motif substitutions to analogous and heterologous sidechains do not affect MHC Class I binding, but do prevent recognition by specific CD8^+^ T cells (Hsu et al. 2012). Thus, epitope mutations may alter the cellular immune response not only by precluding MHC binding, but also by affecting TCR binding to the MHC–peptide complex, a key limitation in considering only MHC–peptide binding affinity when mapping T-cell epitopes. It would be instructive to characterize *in silico* how TCR binding differs between reference and variant epitopes predicted by this study. Moreover, MHC–peptide binding prediction models fail to predict all experimentally validated T-cell epitopes; thus, it is possible that some amino acid variants not predicted to lie within epitope sites could, in fact, be epitope escape variants.

Additionally, while IFN-γ secretion is a widely used proxy for CD8^+^ T-cell activation (Slifka and Whitton 2000, Bergamaschi et al. 2021, Metaxaki et al. 2025), we recognize that PBMCs contain multiple cell populations capable of producing IFN-γ following stimulation, including NK cells (Paolini et al. 2015), monocytes (Kraaij et al. 2014), and other T-cell subsets (Kumar 2017). The peptides used in this study were restricted to MHC Class I alleles; thus, they are expected to preferentially activate CD8^+^ T cells through the Class I presentation pathway. As these represent peptide-specific memory responses, the majority of IFN-γ detected is likely to be T-cell-derived. However, it remains possible that T-cell activation induces secondary cytokine release from other IFN-γ-producing cells within the PBMC population, especially those antigen-presenting cells which would be closest in proximity to activated T cells. Previous work using the same PBMC system demonstrated that CD8^+^ T cells are the primary, though not exclusive, source of IFN-γ under these assay conditions (Krishna et al. 2024), leading us to conclude that the IFN-γ response seen here is primarily but not entirely driven by CD8^+^ T cells within the PBMC population.

## Conclusions

In conclusion, candidate HAdV-F epitopes can successfully be identified *in silico* and validated *in vitro*. Some predicted epitopes are highly conserved among HAdVs, while others are type-specific and show intratypic variation. The results of this study illustrate the importance of jointly considering the genetic diversity of viruses and their hosts. It appears that HAdV-F is unable to mutate to escape all common HLA alleles in a given region, so vaccine candidates designed to elicit cytotoxic T-cell responses may offer a promising strategy for HAdV-F control.

## Supplementary Material

R1_Enteric_AdV_supplementary_071125_veaf098

AdV_F_Tables_Appendices_veaf098

## Data Availability

The immunology data that support the findings of this study are available on request from the corresponding author. The data are not publicly available due to privacy or ethical restrictions. Where not subject to privacy or ethical restrictions, the data that support the findings of this study are available in the supplementary material of this article.
