# Supplementary material for: Viral diversity influences T-cell responses to enteric human adenoviruses F40 and F41
Source: Virus Evol. 2025 Dec 18;12(1):veaf098. doi: 10.1093/ve/veaf098 (PMC12821363; doi:10.1093/ve/veaf098)
Supplement: R1_Enteric_AdV_supplementary_071125_veaf098 [file r1_enteric_adv_supplementary_071125_veaf098.pdf]

Title: Viral Diversity Influences T cell Responses to Enteric Human Adenoviruses F40/41

Short title: F40/41 diversity and T cells responses

Authors

Holly M. Craven*, Jennifer P. Hoang*, Rookmini Mukhopadhyay, Arnold W. Lambisia, Benjamin A. C. Krishna, Benjamin J. Ravenhill, Charles N. Agoti, Charlotte J. Houldcroft

Supplementary figures

Supplementary figure 1

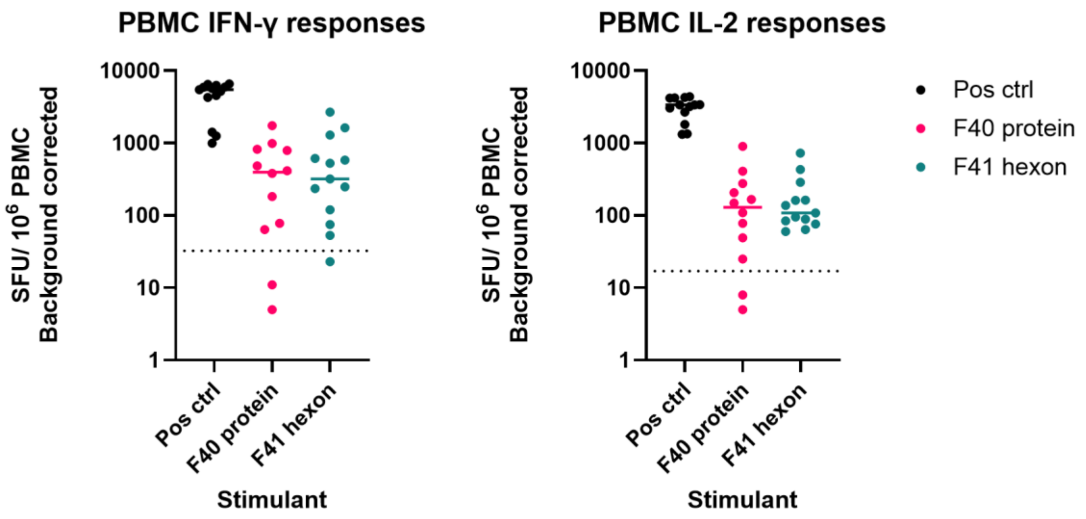

Supplementary figure 1 legend

Analysis of AdV specific IFN-γ and IL-2 FluoroSpot responses to an AdV peptide pool (F41) and whole virion lysate (F40) in a cohort of healthy blood donors, quantified by Fluorospot. The positive control was polyclonal stimulation with anti-human CD3/CD38 antibodies (Immunocult; StemCell). The negative control was stimulation with TexMacs media containing DMSO; responses to the negative control were deducted from each well (background correction). Responses calculated as spot-forming units (SFU) per 10e6 PBMC (background corrected). F41 responses for 10/13 donors previously published (1). The dotted lines indicate the boundary between a positive and a negative response. There was no statistically significant difference in the frequency of responses to F40 protein lysate and F41 hexon peptide pool (two way ANOVA).

Supplementary figure 2

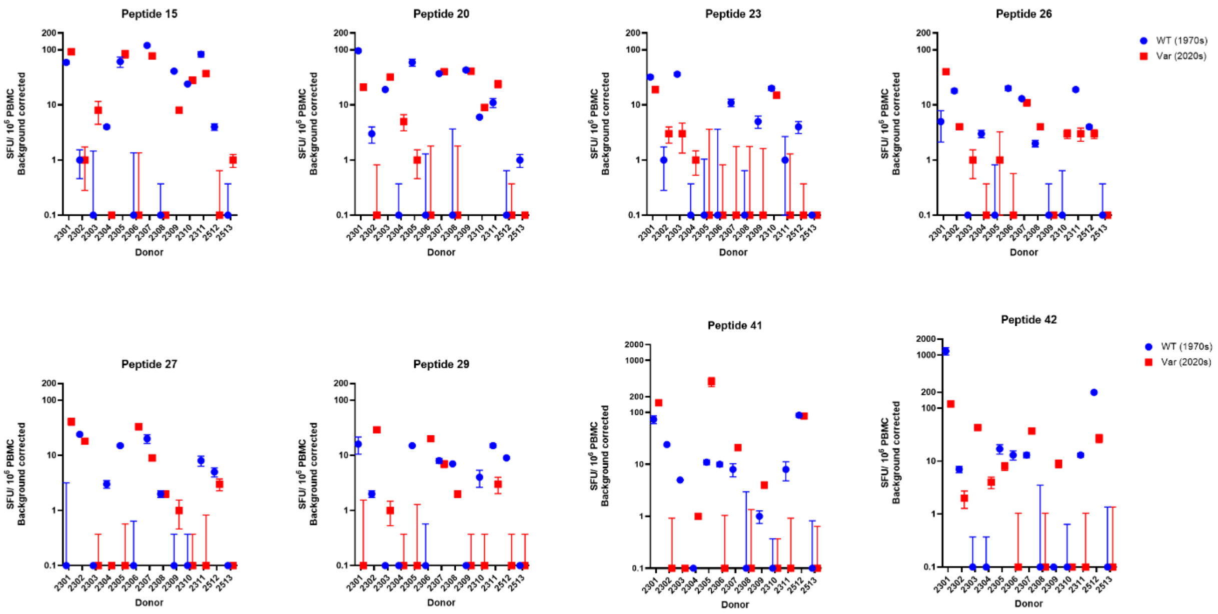

Supplementary figure 2 legend

Analysis of paired within-donor PBMC IFN-γ responses to F41 hexon individual peptide stimulation. PBMC from healthy blood donors were stimulated in triplicate with individual synthetic peptides representing predicted epitopes within the F40 hexon protein which have mutated between the 1970s and 2019-2022. Mean IFN-γ responses are presented for each pair of peptides for each donor. WT: wild type 1970s reference sequence; Var: 2019-2022 variant sequence. Responses are calculated as spot-forming units (SFU) per 10e6 PBMC (background corrected) and shown with SEM.

Supplementary methods

<u>Average Nucleotide Identities</u>

Average nucleotide identities were calculated for the nucleotide sequences of the hexon, penton, short fiber and long fiber genes of HAdV-F40 (NC_001454.1) and F41 (DQ315364.2) using the EZBiocloud ANI calculator (2).

<u>Phenotyping</u>

10^5 total PBMC was stained with the following phenotyping cocktail, containing 2 µL of each antibody: anti-CD3—fluorescein isothiocyanate (FITC), clone UCHT1; anti-CD4—phycoerythrin (PE), clone RPA-T4; anti-CD8a-peridinin-chlorophyll protein—cyanine 5.5 (PerCP Cy5.5), clone RPA-8a (all BioLegend, London, UK), LIVE/DEAD Fixable Far Red Dead Cell Stain Kit (Thermo Fisher Scientific). Phenotyping and analysis were performed on the BD Accuri C6 and NXT Thermo Fischer Attune NxT flow cytometers.

Acknowledgements

This research was supported by the Cambridge NIHR BRC Cell Phenotyping Hub and the University of Cambridge School of Biological Sciences Flow Cytometry facility.

References

1. Mukhopadhyay R, Lambisia AW, Hoang JP, Ravenhill BJ, Agoti CN, Krishna BA, et al. Adenovirus-Specific T Cells in Adults Are Frequent, Cross-Reactive to Common Childhood Adenovirus Infections and Boosted by Adenovirus-Vectored Vaccines. J Med Virol [Internet]. 2025 Feb 1 [cited 2025 Mar 12];97(2):e70222. Available from: https://onlinelibrary.wiley.com/doi/full/10.1002/jmv.70222

2. Yoon SH, Ha S min, Lim J, Kwon S, Chun J. A large-scale evaluation of algorithms to calculate average nucleotide identity. Antonie van Leeuwenhoek, International Journal of General and Molecular Microbiology [Internet]. 2017 Oct 1 [cited 2025 Sep 4];110(10):1281–6. Available from: https://link.springer.com/article/10.1007/s10482-017-0844-4
